# Multi-lifecycle Assessment of Close-loop Recyclable Wind Turbine Blades

**DOI:** 10.1007/s42824-025-00211-7

**Published:** 2026-01-26

**Authors:** Kyle Pender, Filippo Romoli, Jonathan Fuller

**Affiliations:** 1 University of Bristol, Beacon House, Queens Rd, Bristol, BS8 1QU UK; 2 National Composites Centre, Bristol and Bath Science Park, Bristol, BS16 7FS UK

**Keywords:** Net zero, Wind energy, Wind blade recycling, Lifecycle assessment, Circular economy, Circularity

## Abstract

While over 80% of materials in modern wind power installations are recyclable, the sector continues to grapple with the absence of effective, scalable, and environmentally sustainable methods for managing end-of-life wind turbine blades. Addressing the environmental impact of these blades requires not only the integration of lower-impact materials into the manufacturing process but also the advancement of recycling technologies that enable the creation of high-quality secondary materials. Such initiatives are crucial for facilitating the wind energy sector’s transition towards a circular economy. This study utilises lifecycle assessment to assess the environmental impact linked with the production of recycled glass fibre and recycled thermoplastic (polymethyl methacrylate, PMMA) products derived from composite wind blade waste. The assessment considers thermolysis recycling of thermoplastic-based blade waste, encompassing the subsequent refinement of recovered organics into usable secondary monomers. The process also involves the production of virgin-quality secondary glass fibre products, by cleaning and remelting of glass fibre recyclates. It was determined that recycled thermoplastic and glass fibre products can reduce most environmental impact indicators compared to virgin counterparts and are able to diminish material production global warming potential by up to 54% and 26% respectively. Consequently, wind blades manufactured using recycled thermoplastic and glass fibres are projected to have a 27% lower cradle-to-grave global warming potential compared to blades made with conventional epoxy resin and virgin glass fibre reinforcements. As such, both material and recycling solutions demonstrate the potential for decarbonising production and progressing toward a circular economy for wind blades.

**Supplementary Information:**

The online version contains supplementary material available at 10.1007/s42824-025-00211-7.

## Introduction

Industrialised nations are increasingly relying on the expansion of renewable energy sources to achieve net-zero emissions, with wind energy positioned as a central driver of the global shift towards green energy. In 2021, wind power generation grew by nearly 273 TWh, the largest increase among all power generation technologies (IEA, “Wind Electricity” 2022). Although wind energy enables electricity production with only 1–3% of the greenhouse gas (GHG) emissions of traditional fossil fuel sources (Schlömer, et al. 2014), there is still significant potential for optimisation within the industry to further accelerate progress towards global net-zero emissions targets.

Numerous studies have employed lifecycle assessment (LCA) to evaluate the environmental impact of wind turbine power plants, consistently finding that raw material production during manufacturing is the primary contributor to GHG emissions in wind energy systems (Bonou et al. 2016; Ghenai 2012; Pender et al. 2024a). While over 80% of materials in current wind power plants are technically recyclable (Khalid et al. 2023), the industry still faces a critical challenge in developing circular, scalable, and low-impact disposal solutions for end-of-life wind turbine blades (WTBs). Research has shown the potential environmental gains achievable through WTB recycling (Pender et al. 2023, 2024b; Pender and Yang 2024). These findings underscore two priority areas for advancing sustainability across the WTB lifecycle: 1) employing materials with a lower environmental footprint and enhanced sustainability in WTB construction, and 2) establishing efficient recycling methods that minimise the environmental impact of WTB disposal.

A substantial body of research has focused on enhancing recycling processes for fibre-reinforced thermoset polymer (FRP) composites used in current WTBs. This work has led to the development of diverse recycling methods at varying levels of technological maturity (Pender and Yang 2023; Karuppannan Gopalraj and Kärki 2020; Rybicka et al. 2016; Ahrens et al. 2023). Broadly, these methods can be divided into three main categories: thermal, mechanical, and chemical processes. Each approach is designed to recover either materials or energy from FRP waste, aiming to replace raw material production or reduce overall energy consumption.

Glass fibres (GF) reinforcements make up the largest mass contribution to typical current WTB designs (Mishnaevsky et al. 2017) and therefore present the greatest opportunity to increase circularity of WTBs. There are three critical technical barriers which limits the market demand for recycled GF (rGF): 1) rGF are discontinuous which relegates their use to low performance, low value applications, 2) rGF strength is significantly degraded compared to virgin GF counterparts (Thomason et al. 2020; Pender and Yang 2020, 2019), and 3) rGF fluffy architecture makes handling and forming challenging compared to virgin counterparts (Karuppannan Gopalraj and Kärki 2020; Pender 2018). These technical barriers are compounded by the relatively low cost of virgin glass fibre (vGF), therefore there is not a strong economic driver to use rGF in their current state. All these factors mean that closed-loop recycling of GF (e.g., blade-to-blade GF recycling) has yet to be demonstrated for WTBs. Without intervention, future generations of WTBs will continue to rely on vGF materials, and function within a linear economy. However, it is possible to use waste GF as a feedstock in the production of secondary glass fibre products, by remelting to produce “virgin-like” rGF. This has the potential to overcome the challenges associated with rGF reuse in WTBs and is an opportunity to move WTBs toward a circular economy.

Thermosetting laminate resins, typically epoxy or polyester (Mishnaevsky et al. 2017), constitute the second most significant material contribution in WTB structures, presenting a crucial opportunity to enhance WTB circularity. Due to their molecular crosslinking, recycling thermosetting resins poses challenges, with many recycling techniques opting to utilise the resin fraction in FRP directly as heat in thermal recycling processes or reclaiming for use as fuel products. An alternative approach involves designing the next generation of WTBs with resin systems that are more readily recyclable and capable of generating secondary polymer products at End-of-Life (EoL). In partnership with Aditya Birla Advanced Materials, Siemens Gamesa has pushed the development of a new resin system (Recyclamine) used in WTBs, culminating in the development of Siemens Gamesa’s RecyclableBlades (Stecher and Salgado 2023). In 2022, Siemens Gamesa installed the first 81-m recyclable blades at RWE’s Kaskasi offshore wind farm (Stecher and Salgado 2023), and the ZEBRA consortium announced LM Wind Power’s successful production of a 62-m recyclable blade prototype using Arkema’s Elium® resin (Nehls 2022). In 2023, MingYang Smart Energy introduced a 75-m blade made with over 95% recyclable materials, becoming the first Chinese OEM to provide recyclable blade technology (Wood 2023).

Infusable polymethyl methacrylate (PMMA) thermoplastic (TP) resins are of particular interest, as they offer comparable performance and manufacturing methods to traditional thermosetting resins (Carnicero et al. 2023, 2022). Additionally, they can be recycled back into methyl methacrylate (MMA) monomers, enabling closed-loop use in subsequent generations of WTBs and facilitating a circular economy for WTBs (Carnicero et al. 2023, 2022; Cousins et al. 2019). Examples of infusable PMMA products include Elium®, produced by Arkema S.A (Nehls 2022) and AKELITE, patented by the Institute of Polymer Science and Technology (ICTP-CSIC) (Carnicero et al. 2023).

This study employs LCA to evaluate the environmental impact of producing secondary GF and PMMA products derived from EoL WTB waste, and systematically compares the environmental impact of these secondary products with their virgin counterparts. The assessment considers thermolysis recycling of TP-based WTB waste, involving the subsequent refinement of recovered organics into usable monomers. Additionally, the process includes downstream cleaning and remelting of the collected GF fraction to create as new rGF products. Both material and recycling solutions demonstrate the potential for establishing closed-loop recycling systems for WTBs. Therefore, LCA was employed to evaluate the impact of using secondary GF and TP products in the production of subsequent generations of WTBs. Simultaneously, a material circularity assessment was performed to quantify the extent to which the evaluated material and recycling strategies support the transition toward a circular economy.

## Materials and Methods

This study was conducted in accordance with the methodologies outlined in the ISO 14040 series of international standards for LCA studies.

### Goal and Scope

The purpose of the LCA is to analyse the environmental impact of incorporating circular materials in WTB structures during their primary use, and to examine how the subsequent recycling and reuse of these materials affects the environmental impact of subsequent WTBs that incorporate recycled materials. The following circular material solutions have been included:

- Infusable PMMA TP as a more readily recyclable solution to standard thermosetting systems.
- Recycled and remelted GF to replace standard virgin GF.

Alongside the LCA, it is the aim of this study to quantify the circularity of WTB scenarios using the Material Circularity Indicators (MCI) methods. By combining both assessment methods, this work aims to evaluate both the environmental impact of WTB designs and the extent to which they enable a transition toward a circular economy.

#### Functional Unit

The primary function of a wind power plant is electricity generation. This LCA focuses exclusively on the WTB, with all other components excluded from the analysis. For simplicity, it is assumed that the use of virgin or recycling materials in the WTB does not affect the wind power plant capacity or annual/lifetime energy production. As such, the function of the WTB is unaffected across the scenarios assessed, and the functional unit (FU) for this LCA is: The production of a WTB with any of the designs described in the investigation. As such, the FU coincides with the reference flow which is one WTB.

#### Scenarios

**Fig. 1:**
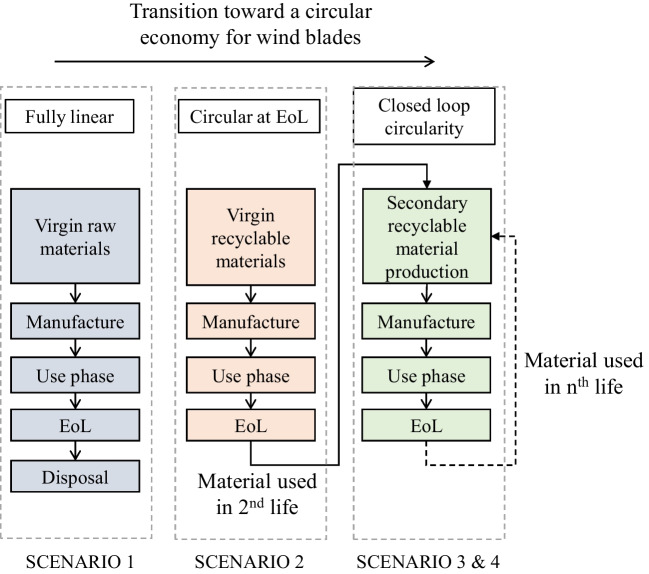
Illustration of how scenarios selected enable the assessment of transitioning from a linear to circular economy for WTBs

Scenarios have been selected to assess the cradle-to-grave impact of WTB designs, considering the shift from a linear economy to a closed-loop circular economy for WTBs, as is illustrated in Fig. 1. To that end, several WTB design, laminate resin systems, and GF reinforcements which can enable this transition were evaluated.

##### Laminate resin scenarios

Three laminate resin scenarios were considered:

1. *Epoxy resin* – Bisphenol A epoxy resin system widely used in production of wind blade structures.
2. *Virgin TP (vTP)* – Infusable acrylic TP resin offering comparable performance to thermosetting resins, allowing the products and systems that use it to be more readily recycled (Cousins et al. 2019).
3. *Recycled TP (rTP)* – Infusable TP product recycled from EoL PMMA based WTB using thermolysis recycling and organics refining process into secondary MMA monomers. This is chemically the same as vTP and can undergo closed loop thermolysis recycling at EoL.

##### GF Reinforcement Scenarios

Three GF reinforcements scenarios were considered:

1. *vGF* – Virgin E-glass fibre widely used in production of wind blade structures.
2. *rGF_50%* – rGF produced with 50 wt.% recycle GF content recycled from EoL WTB using thermolysis process followed by oxidative cleaning and remelting into secondary GF product.
3. *rGF_100%* – Same as “rGF_50%” above but produced with 100 wt.% recycle GF content.

##### WTB Design Scenarios

Two different WTB designs were considered:

1. *GF WTB*: A WTB design reinforced solely with GF, characteristic of smaller-scale WTB material compositions.
2. *GF/CF hybrid WTB*: A hybrid WTB design featuring GF reinforcement in the shells and shear webs, with carbon fibre (CF) reinforcement in the spar caps, representative of larger-scale onshore and offshore installations.

##### Combined Scenarios

By combining the various material scenarios, a total of four scenarios were assessed which were applied to both WTB designs, which are described in Table 1.

- *Scenario 1: Baseline* – Representative of WTB manufactured using industry standard virgin raw materials.
- *Scenario 2: vTP/vGF* – Representative of current generation of “recyclable” WTBs manufactured using virgin materials (vTP, vGF).
- *Scenario 3: rTP/rGF_50%* – Representative of next generation of “recyclable” WTBs manufactured using rTP and rGF with 50% recycled GF content in the melt.
- *Scenario 4: rTP/rGF_100%* – Representative of next generation of “recyclable” WTBs manufactured using rTP and rGF with 100% recycled GF content in the melt.

**Table 1:** Description of various scenarios investigated

		*Scenario 1* Baseline	*Scenario 2* vTP/vGF	*Scenario 3* rTP/rGF_50%	*Scenario 4* rTP/rGF_100%
Blade design		GF WTB			
		GF/CF hybrid WTB			
Materials	Laminate resin	Epoxy resin	vTP	rTP	
	Glass fibre	vGF		rGF	
	rGF content in remelt	N/A		50 wt.%	100 wt.%
EoL method(s)		Landfill	Thermolysis recycling		

The EoL treatment method used is critical in determining the WTB circularity and therefore must be considered when evaluating the MCIs. For the Scenario 1 (Baseline) it is assumed that the WTB is landfilled whereas, due to the use of recyclable TP, it is assumed that Scenario 2–4 are recycled via thermolysis recycling.

#### System Boundary

**Fig. 2:**
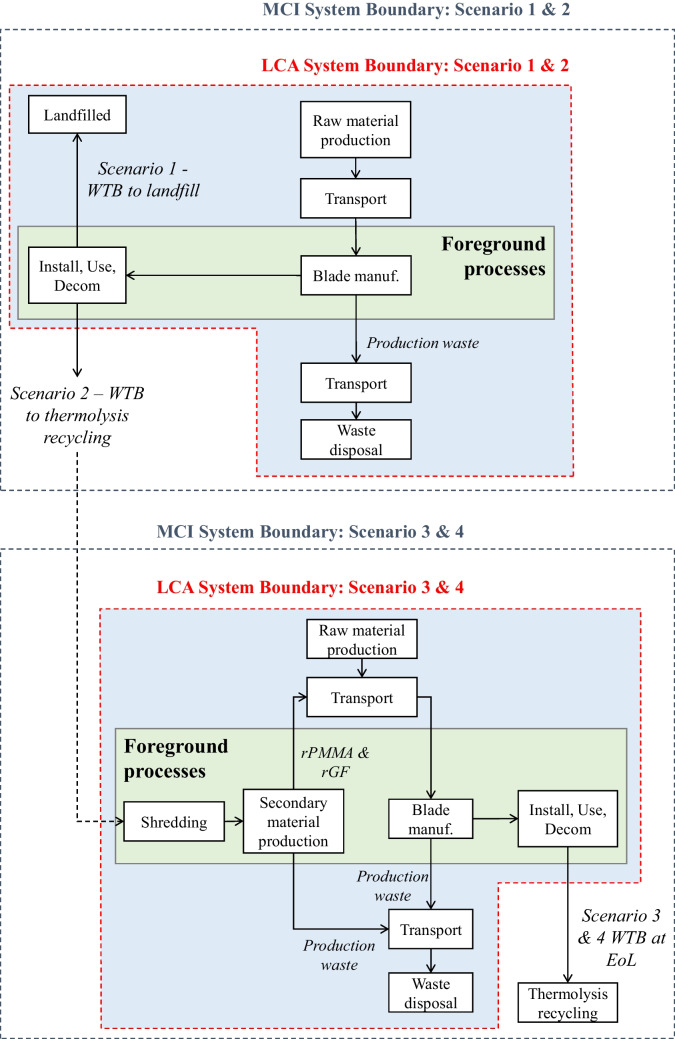
System boundary of LCA and MCI analysis showing foreground and background processes

Figure 2 shows the system boundaries of the LCA and MCI analysis. The LCA and MCI system boundaries are the same for Scenario 1 and slightly different for Scenarios 2 to 4.

The LCA employs the cut-off approach (Frischknecht 2010). For Scenario 1, where the WTB is disposed of at EoL, disposal processes fall within the system boundary, and their impacts are attributed to the WTB. In contrast, for Scenarios 2 to 4, where the WTB is recycled at EoL, the subsequent use of materials assumes the environmental impacts associated with collection, recycling, and disposal of recycling by-products. Secondary materials are not assigned any environmental burdens from the primary production processes.

MCIs require consideration of the full product lifecycle (regardless of EoL treatment) to quantify restorative and non-restorative material flow in the WTB production and EoL phases. The system boundary for the MCI analysis is therefore consistent across scenarios.

Foreground processes were modelled to generate data used in the LCA and MCI analysis, whereas background processes utilised either aggregated dataset or data sourced from literature.

### Processes

**Fig. 3:**
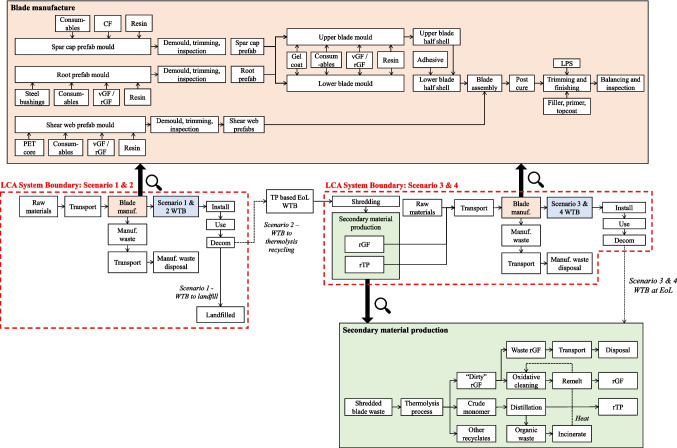
Processes considered in the scenarios

The LCA considered all major processes within the WTB lifecycle phases: manufacture, installation, use, decommissioning, disposal/recycling. Figure 3 maps the processes considered in the analysis and details the steps involved in WTB manufacturing and recycling. The processes involved in manufacture, installation, use, and decommissioning are described in the Authors’ previous publication (Pender et al. 2024c).

#### Thermolysis Recycling

Thermolysis recycling involves the application of heat to break down complex organic materials into simpler components. In the context of polymers, thermolysis recycling refers to the thermal degradation of these materials to produce valuable products such as monomers. The process involves heating the material in the absence of oxygen to prevent combustion. Upon exposure to elevated temperatures, chemical bonds within the polymer chains break, leading to the decomposition of the polymer into smaller molecules. The resulting products can be further processed and refined for various applications.

Shredded PMMA based WTB waste is introduced in a continuous reactor for thermolysis at 400 °C. Upon heating, the PMMA is depolymerised to MMA. Other polymers (e.g., adhesives, surface coatings, foam cores) are decomposed and contaminate the MMA monomers which requires subsequent refining to extract pure MMA. So called, “Dirty” GF and “Dirty” CF are liberated from the PMMA matrix which have non-decomposed polymeric surface residues which require cleaning prior to reuse. Metallic components (such as steel root bushings and aluminium/copper lightening protection systems) can be isolated from the shredded WTB waste using standard ferrous and non-ferrous metallic separation techniques and processed via traditional metallic recycling routes.

#### rTP Production

The gaseous products from WTB thermolysis are condensed to produce crude MMA monomers – a liquid containing both MMA products from PMMA thermolysis and decomposition products from other organic components of the WTB. This crude MMA monomer is immediately subjected to a continuous distillation for purification to obtain a > 99% purity MMA ready for being introduced in the formulation of new rTP, having the exact same properties as virgin PMMA. Non-MMA components are isolated during distillation, then condensed, and incinerated to provide heat to the oxidative rGF cleaning step described below.

#### rGF Production

To facilitate closed-loop recycling of GF into WTB production, this study explored the use of GF recovered from WTB waste as feedstock in a melt furnace, producing rGF products with equivalent performance and form to vGF counterparts. Prior to remelting, the “Dirty” GF must be thermo-oxidatively cleaned to remove organic surface contaminants utilising excess heat produced during incineration of the liquid waste organics. Clean GFs are then milled prior to feeding into the melt furnace along with other raw materials in the production of rGF product.

### Lifecycle Inventory

#### Data Sources

Data/assumptions used to generate the LCI data for foreground processes are described in Sect. 2.3.2 below. LCI secondary datasets from Sphera Managed LCA Content Database (formerly GaBi Database) 2022 were used for background processes.

#### Assumptions in LCI Calculations

##### WTB design

The GF WTB design applied in this study is derived from the 5 MW reference blade model reported by the National Renewable Energy Laboratory (NREL) (Jonkman et al. 2009). For the GF/CF hybrid WTB, the design is adapted from the 15 MW reference turbine specified by the International Energy Agency (IEA). The bill of materials (BoM) detailing the compositions for both the GF and GF/CF hybrid WTB designs is provided in Table S1. It was assumed that epoxy resin, vTP, and rTP could be used interchangeably without impacting WTB design, therefore the same BoM was used across Scenarios 1 to 4.

##### WTB Manufacture, Installation, Use and Decommissioning

All assumption made with respect to the WTB manufacture, installation, use and decommissioning phases are detailed in the Authors’ previous publication (Pender et al. 2024c).

##### WTB Recycling/Secondary Material Production

It is assumed that the WTB waste goes through shredding and granulation prior to thermolyzing. The energy demand required to conduct the shredding was estimated to be 0.11 MJ/kg WTB based on energy demand data provided by a supplier of industrial shredding equipment. It is assumed that energy consumption during granulation is 0.27 MJ/kg of blade waste based on granulator primary energy data reported in Shuaib and Mativenga (2016) when processing similar composite material.

Primary data for thermolysis recycling of TP based WTB waste was collected by Arkema S.A. using an electrically heated pyrolysis unit described in Bel et al. (2021). Data captured included the energy consumption to maintain the required processing temperature, energy requirements for cooling during condensation, the mass and composition of off gases, and the mass of output products (fibrous fraction, condensed organics).

It is assumed that all electrical and thermal energy input during WTB recycling is supplied from the UK national electrical grid and gas pipelines respectively.

##### rTP Production

Arkema S.A. collected primary energy and material flow data for the distillation separation process required to isolate the rTP monomers from the other contaminant organics. Heat was supplied to distillation process in the form of steam generated from natural gas and removed via a cooling system to enable condensation of the products. Superlight gases (CO_2_, N_2_, methanol) are produced during the thermolysis/distillation process which are assumed to be directly emitted to the atmosphere. The composition of the waste liquid organics is dependent on the polymer fractions in the WTB and was evaluated by assuming it was composed of all non-PMMA based polymers (adhesive, polyurethane coatings, PET core) in the waste WTB feedstock. It was assumed that the waste liquid organics were oxidised to CO_2_ H_2_O and NOx during incineration and assumed to be directly emitted to the atmosphere. Waste liquid organics were determined to have a calorific value of approximately 25 MJ/kg and the heat from combustion was assumed to be used in the thermal cleaning of the “Dirty” GFs.

##### rGF Production

Material analysis following thermolysis trials determined that the “Dirty” GFs have 3–4 wt.% surface residue which was assumed to be char and fully combusted to CO_2_ during the cleaning stage. The energy required to conduct the thermo-oxidative cleaning was assumed to be supplied by the exothermic combustion of the waste liquid organics and the char itself, as such, no additional external source of energy was required to conduct the GF cleaning.

Primary data for virgin glass melting was collected by Owens Corning during standard production using one of their melting furnaces. Data captured during melting included thermal and electrical energy consumption, direct emissions, and product and waste material flows. Theoretical reductions from the use of recovered GF feedstocks in the place of virgin materials were applied to the model to produce LCI data for the impact to produce 1 kg of rGF from clean recovered GF feedstocks at a variety of waste GF contents.

It is assumed that rTP and remelted rGF products have the same mechanical properties as virgin PMMA and E-glass fibre respectively and can be used as a drop in solution in the production of WTBs without affecting the manufacturing process.

LCI data for the secondary material production are given in Figure 18 and Figure 19.

#### Allocation

The WTB manufacturing is not a multifunctional process; there is only one output therefore no allocation needed to be considered in the study. The impact of all production waste was attributed to the WTB itself.

**Fig. 4:**
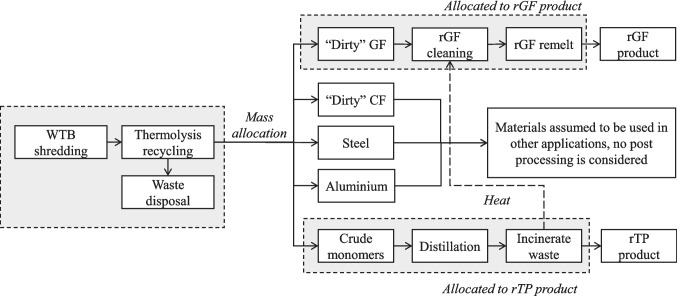
Allocation approach used for secondary material production in Scenario 3 and 4

WTB recycling using thermolysis is a multifunctional process and produces multiple material outputs, shown in Fig. 4. Mass allocation was used to attribute the impact of downsizing and thermolysis processes to each of the product materials. Post processing of the “Dirty” GF and crude monomers is required, and partitioning was used to only apply the impact of these processes to the relevant output products, as is shown in Fig. 4. The waste liquid organics are a byproduct of the rTP production process; therefore, the impact of combustion was attributed to the rTP. By following the cut-off approach for EoL treatment, no burden is attributed to the waste WTB materials entering the system and it is assumed that impacts associated with decommissioning are attributed to the primary WTB lifecycle.

#### Cut-off Criteria

For WTB manufacture and secondary material production no specific cut-off criteria were formally defined for this study. However, the following general exclusions were applied: human energy inputs in processes; production, disposal, and maintenance of infrastructure; employee commuting and business travel; auxiliary functions (such as research and development, marketing, finance, and management); and incidental GHG emissions from equipment like air conditioning and refrigeration units during manufacturing.

### Lifecycle Impact Assessment

The "CML2001—August 2016 update" impact assessment methodology was employed in this study, with a focus on evaluating the following environmental impact indicators:

- Abiotic Depletion (elements) [kg Sb eq.]
- Abiotic Depletion (fossil) [MJ]
- Acidification Potential [kg SO_2_ eq.]
- Eutrophication Potential [kg Phosphate eq.]
- Freshwater Aquatic Ecotoxicity Potential [kg DCB eq.]
- Global Warming Potential (100 years) [kg CO_2_ eq.]
- Global Warming Potential (100 years), excl. biogenic carbon [kg CO_2_ eq.]
- Human Toxicity Potential [kg DCB eq.]
- Marine Aquatic Ecotoxicity Potential [kg DCB eq.]
- Ozone Layer Depletion Potential [kg R11 eq.]
- Photochem. Ozone Creation Potential [kg Ethene eq.]
- Terrestric Ecotoxicity Potential [kg DCB eq.]

### MCI Methodology

**Fig. 5:**
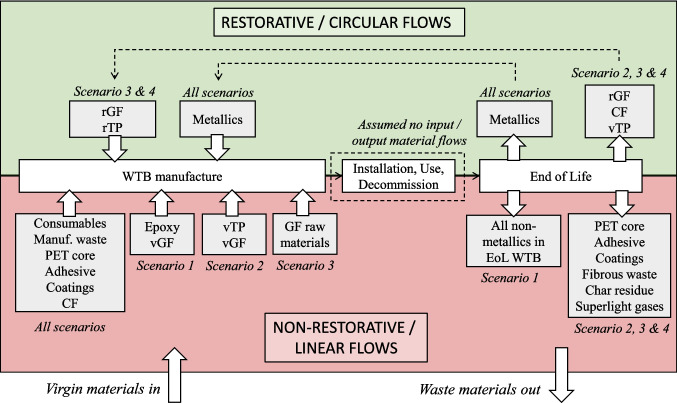
Illustration of input/output materials across WTB lifecycle and categorisation of restorative/non-restorative flows for each scenario

MCIs were calculated based on the methodology in “Circularity Indicators: An Approach to Measuring Circularity”. (2015) and described in a prior publication by the authors (Pender et al. 2023). The MCI quantifies the degree to which the material flows of the WTBs minimise linearity and maximise circularity, evaluating both usage duration and intensity relative to an industry-standard product. The MCI integrates three key factors: 1) the amount of virgin raw material used, 2) the mass of non-recoverable waste attributed to the product, and 3) a utility factor reflecting product longevity and usage intensity. For Scenarios 1 and 2, it is assumed that all raw materials are virgin, while in Scenarios 3 and 4, rTP and rGF are incorporated. The MCI ranges from 0 to 1, where values closer to 1 indicate higher circularity; a fully circular system (MCI = 1) would use entirely restorative feedstock and restore all output materials through reuse or recycling. Figure 5 Illustrates the input/output materials across WTB lifecycle and the categorisation of restorative/non-restorative flows for each scenario when assessing the MCIs.

## Results

### Impact of Baseline WTB Scenarios

**Fig. 6:**
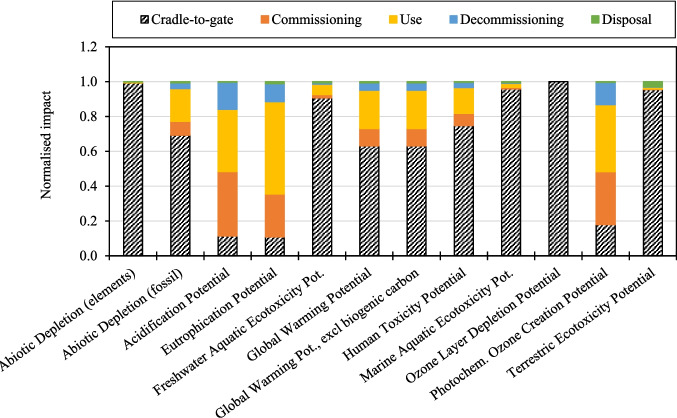
Contributions toward cradle-to-grave impacts for the “Baseline_GF WTB” scenario

**Fig. 7:**
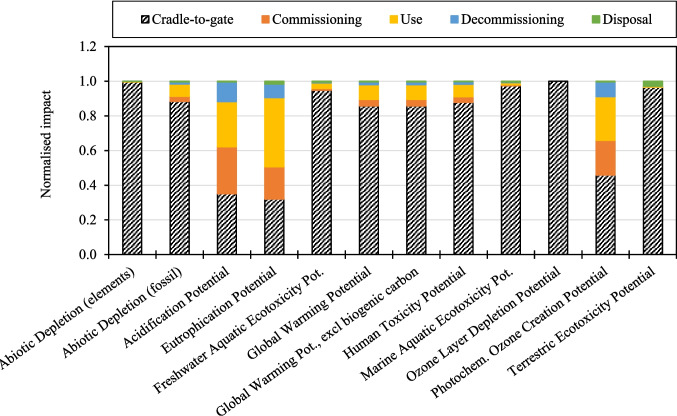
Contributions toward cradle-to-grave impacts for the Baseline_GF/CF hybrid WTB scenario

Figure 6 and Fig. 7 present the contributions toward cradle-to-grave impacts for Scenario 1, “Baseline_GF WTB” and “Baseline_GF/CF hybrid WTB”, scenarios respectively. The relative contributions from the main lifecycle phases are given, where cradle-to-gate includes the production and transportation of raw materials and processes involved in the WTB manufacturing (including production waste disposal). Figure 6 and Fig. 7 show that, regardless of the WTB blade design assessed, the cradle-to-gate is the largest contributor to most impact indictors. The exceptions being Acidification Potential, Eutrophication Potential, and Photochemical Ozone Creation Potential, which are dominated by fuel consumed during commissioning, use, and decommissioning activities. Critically for energy generation decarbonisation, Fig. 6 and Fig. 7 show that the cradle-to-gate phase accounts for 63% and 86% of the GWP of Baseline_GF WTB and Baseline_GF/CF hybrid WTB scenarios respectively. There should therefore be a priority to identify and deploy strategies to reduce the WTB cradle-to-gate impacts (since savings here have the greatest potential to reduce total lifecycle impacts of WTBs).

**Fig. 8:**
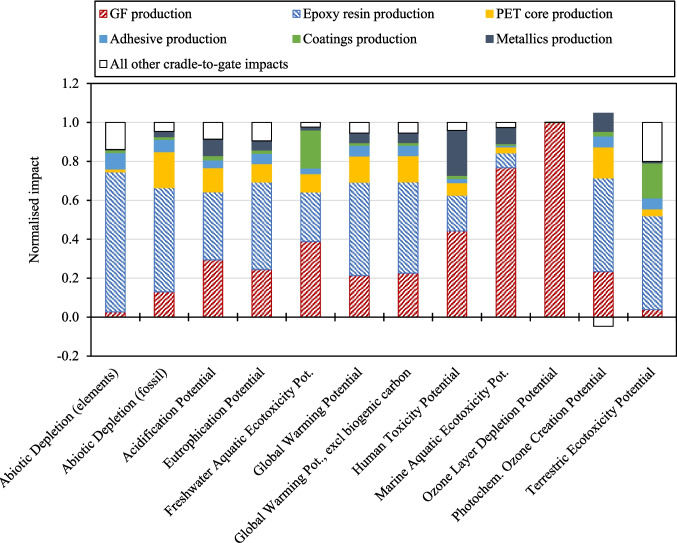
Contributions toward cradle-to-gate impacts for the “Baseline_GF WTB” scenario

**Fig. 9:**
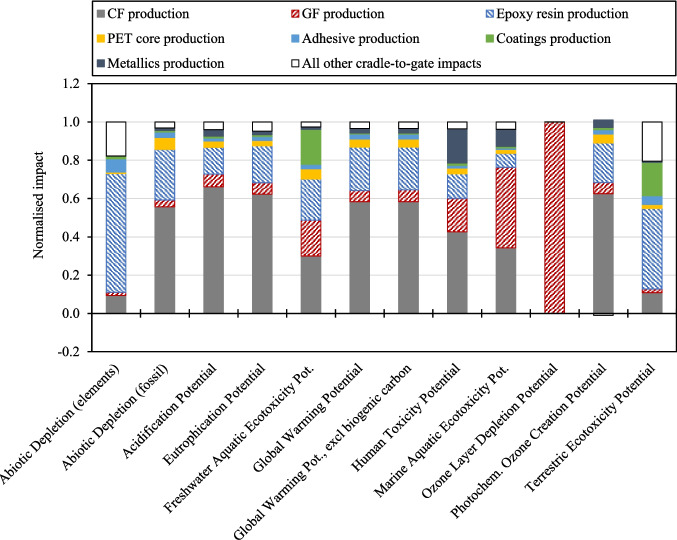
Contributions toward cradle-to-gate impacts for the Baseline_GF/CF hybrid WTB scenario

Figure 8 and Fig. 9 present the contributions toward cradle-to-gate impacts for Scenario 1, “Baseline_GF WTB” and “Baseline_GF/CF hybrid WTB”, scenarios respectively. The relative impacts to produce each of the raw materials used in the WTB are given, alongside “All other cradle-to-gate impacts” which includes the impact from consumable material production, transportation of raw and waste materials, manufacturing and facility processes, and disposal of production waste. Figure 8 and Fig. 9 show that, across all impact indicators assessed, the production of raw materials used in the WTB structure is the largest contributor to the WTB cradle-to-gate environmental impact. In the context of wind energy decarbonisation, Fig. 8 and Fig. 9 show that between 95% and 97% of the GWP attributable to the production of WTBs comes from the upstream production of the raw structural materials. It is therefore critical to identify and deploy lower impact, alternative material solutions in the production of WTBs as means of reducing the lifetime impact of WTBs.

Figure 8 and Fig. 9 show that the highest impact environmental material for each of the WTB designs is dependent on both the impact indicator of interest, as well as the material composition of the WTB. Figure 8 shows that for GF WTBs, the combination of GF and epoxy resin production is the largest contributor to all the WTB impact indicators (in part due to these two materials accounting for 72 wt.% of the WTB BoM). Lower impact solutions to these two materials should therefore be developed to enable the greatest reduction in GF WTB cradle-to-gate impact. On the other hand, Fig. 9 shows that for GF/CF hybrid WTBs, CF production is the majority contributor to several indicators such as Abiotic Depletion (fossil), Acidification Potential, Eutrophication Potential, GWP, and Photochemical Ozone Creation Potential. The production of CF accounts for 58% of the cradle-to-gate GWP of the GF/CF hybrid WTB, therefore lower impact alternatives to CF and/or strategies to reduce the impact of current CF production practices are required to decarbonise WTB designs utilising CF reinforcements. Regardless, GF and epoxy resin production combined accounts for a significant proportion of the environmental impacts of GF/CF hybrid WTBs, therefore lower impact solutions (such as those investigated in this study) should be pursued as part of a holistic approach to minimising wind energy impact.

### Impact of Secondary Materials

#### rTP Product

**Fig. 10:**
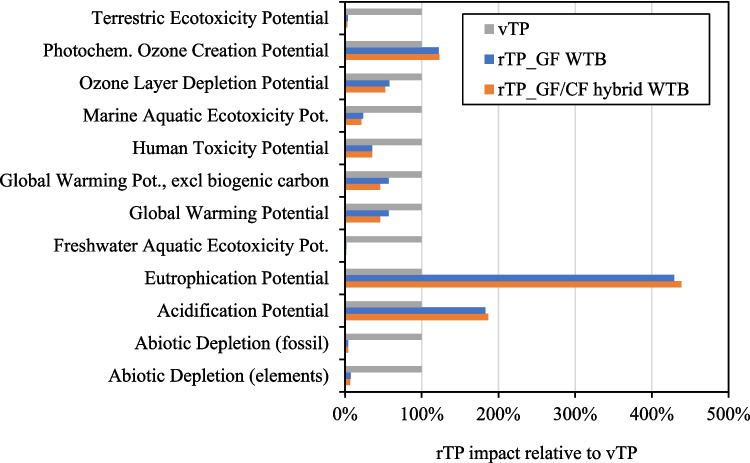
Impact of rTP production from both GF WTB and GF/CF hybrid WTB waste feedstocks

**Fig. 11:**
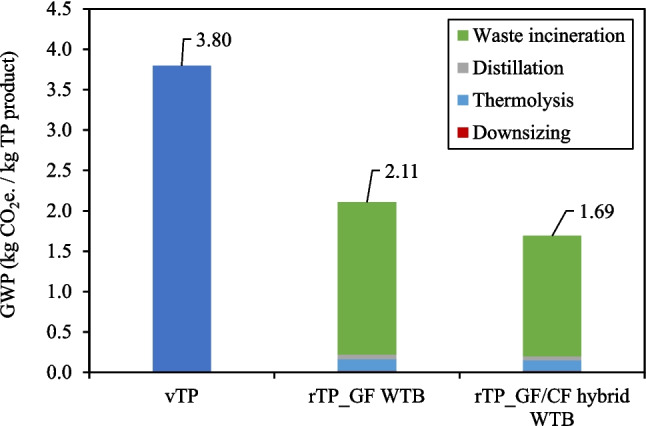
GWP of vTP compared to rTP products from GF WTB and GF/CF hybrid WTB waste

Figure 10 gives the impact of rTP production from both GF WTB and GF/CF hybrid WTB waste feedstocks, relative to the impact to produce conventional vTP. Figure 10 shows that for most indicators, rTP products have a lower impact compared to vTP counterparts which highlights the potential environmental benefits that can be enabled by production and utilisation of rTP. Eutrophication potential and Acidification Potential are however higher for rTP compared to vTP. This is due to NO_x_ produced during the incineration of organic liquid byproduct from the thermolysis and distillation process and could likely be avoided by additional abatement processes not considered in the LCA. rTP produced from GF/CF hybrid WTB waste feedstock has the lowest impact. This is in part, because thermolysis recycling of GF/CF hybrid WTB waste produces an additional CF product which also shares the burden of the downsizing and thermolysis process (which is not present in the GF WTB scenario). Additionally, much of the impacts arise from incineration of the waste organic liquid (as is shown in Fig. 11 for GWP below) and the GF/CF hybrid WTB design has a proportionally lower mass of waste organics requiring incineration compared to GF WTB (due to less PET core material as shown in WTB BoMs in Table S1). Figure 10 shows that rTP products are expected to have between 43% and 54% lower GWP compared to vTP (depending on the recycling feedstock used) which is discussed further in Fig. 11.

Considering the pressing impact of GHG emissions on environmental and ecological stability, it is essential to prioritise GWP as a key impact indicator for detailed analysis. Figure 11 gives the GWP values of vTP compared to rTP products from GF WTB and GF/CF hybrid WTB waste feedstocks, showing that the GWP of rTP is 1.7–2.1 kg CO_2_e./kg lower than conventional vTP (depending on the recycling feedstock used). The GWP for vTP in Fig. 11, from which the rTP products are compared, is calculated based on LCI aggregated data produced by Sphera. Arkema S.A. have produced LCA impact data for their infusable PMMA product “Elium ® 191 O/SA”, which purports to have a production GWP of 3.6 kg CO_2_ e./kg Elium, and is therefore closely aligned with the vTP data point in Fig. 11 (Arkema 2021).

Figure 11 shows that the majority of the GWP of rTP products is the GHG emissions produced during waste incineration. A strategy for further reducing the GWP of rTP products should therefore focus on methods to utilise the non-MMA monomer fraction of the organics to avoid/mitigate waste incineration and the direct GHG emissions thereof.

#### rGF Products

**Fig. 12:**
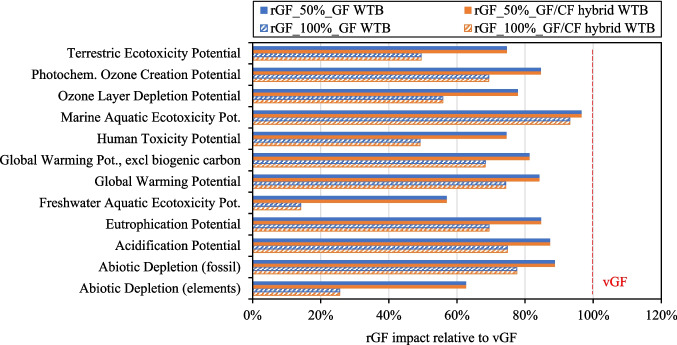
Impact of rGF production from both GF WTB and GF/CF hybrid WTB waste feedstocks

Figure 12 gives the impact of rGF production relative to the impact to produce vGF for 1) GF WTB and GF/CF hybrid WTB waste feedstocks and 2) 50 wt.% and 100 wt.% waste GF content in the remelt. Figure 12 shows that for all indicators, rGF products have a lower environmental impact compared to vGF counterparts, which highlights the potential environmental benefits that can be enabled by production and utilisation of rGF produced using the described recycling method. Additionally, the remelt rGF products assessed in Fig. 12 have matched mechanical performance and continuous filament format as vGF and can therefore be considered a true drop in solution to replace vGF in high demanding applications such as WTB structures.

**Fig. 13:**
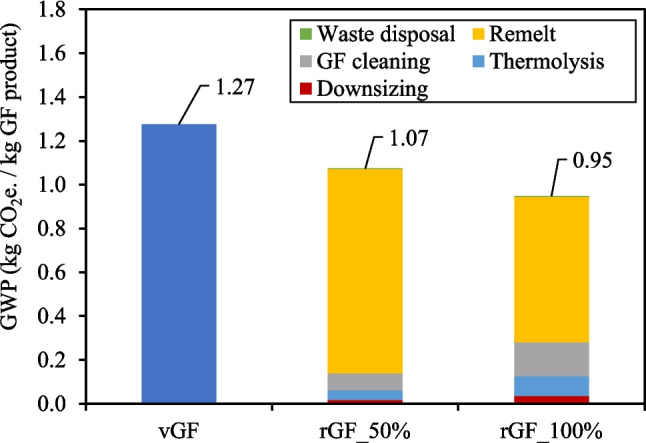
GWP of vGF compared to rGF_50% and rGF_100% products from GF WTB waste

Figure 12 shows that, across the two waste WTB feedstocks assessed, the waste composition does not significantly affect the resulting environmental impact of the rGF product. This is because most of the impact to produce the remelted rGF products comes from the remelting phase (this is discussed in Fig. 13 for GWP specifically), which is independent of upstream processes required to produce the clean waste GF remelt feedstock.

Figure 13 gives the GWP values of vGF compared to rGF_50% and rGF_100% products from GF WTB waste. It is important to highlight that the LCI data used to generate the impact of both 1) the vGF product and 2) the remelt phase of the rGF products, was based on actual and theoretical data for the same Owens Corning production facility/technology, and therefore allows for a fair comparison to be made across product scenarios. Figure 13 shows that the GWP of rGF_50% and rGF_100% products is between 16% and 26% lower than the conventional vGF product respectively.

Figure 13 shows that the majority of the GWP of rGF products is attributable to the GHG emissions produced during the remelt phase. Critically however, the GWP of the remelting phase to produce rGF products remains lower than the GWP of vGF products, due to 1) the complete/partial mitigation of raw material inputs and 2) less energy intensive operating conditions during the melt phase (remelting rGF requires less energy than an equivalent mass of raw materials). Regardless, the remelting remains energy intensive and utilises natural gas to achieve and maintain the required melt temperature. As such, decarbonisation strategies of both vGF and remelted rGF products should focus on 1) electrification using green energy sources where possible, and 2) utilising decarbonised fuel source where electrification is not possible.

### Impact of Alternative WTB Scenarios

**Fig. 14:**
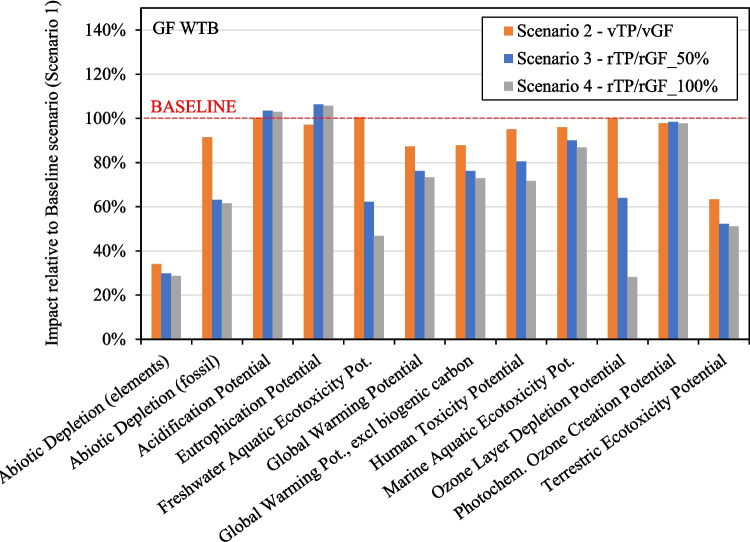
Cradle-to-gate impact of Scenarios 2, 3 & 4 relative to Baseline for GF WTB scenario

**Fig. 15:**
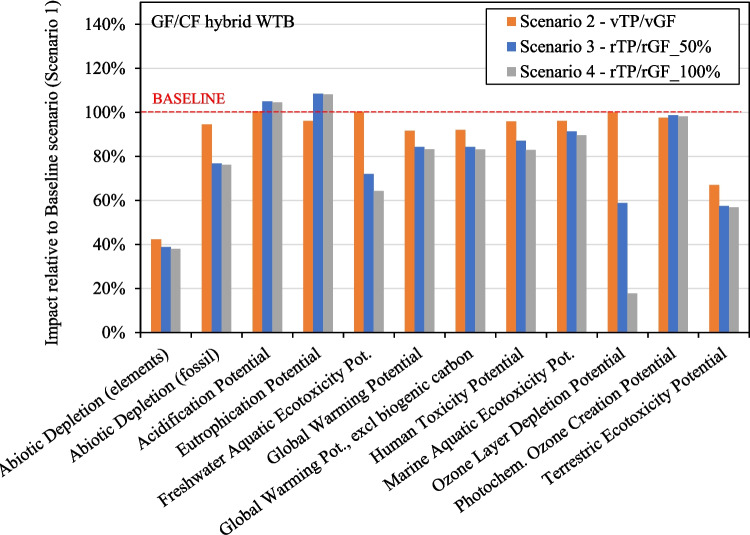
Cradle-to-gate impact of Scenarios 2, 3 & 4 relative to Baseline for GF/CF hybrid WTB scenario

Figure 14 and Fig. 15 present the cradle-to-grave impact of Scenarios 2, 3, and 4 compared to the Baseline for both WTB design scenarios. These figures show that each of the alternative material scenarios (Scenarios 2, 3 & 4) exhibits a lower impact than the baseline (Scenario 1), across most impact indicators considered. The exceptions to this are Acidification Potential and Eutrophication Potential for reasons discussed in Fig. 10 above.

Of significant importance for the decarbonisation of wind energy, Fig. 14 and Fig. 15 demonstrate that substituting the standard epoxy resin system with vTP (Scenario 2) can reduce the cradle-to-grave GWP. Although Scenario 2 still relies entirely on virgin raw materials during production, the utilisation of vTP could serve as a step in reducing the cradle-to-gate GWP of currently produced WTBs while enabling the development of lower-impact WTBs in the future. This could be achieved by facilitating the production of secondary materials at a reduced environmental impact (as shown in Fig. 11), that critically, can be used back in WTB production and to reduce the cradle-to-gate GWP of subsequent WTB structures.

This is evident in Fig. 14 and Fig. 15, where the incorporation of rTP and rGF in WTB structures leads to a further drop in GWP for Scenarios 3 and 4. This is expected to reduce the cradle-to-grave GWP by up to 27% and 16% (compared to baseline) for GF WTB and GF/CF hybrid WTB scenarios respectively. The utilisation of rTP and rGF holds a relatively greater potential for reducing the GWP of GF WTBs as opposed to GF/CF hybrids, given that the GWP of GF/CF hybrids is dominated by the production of CF. Regardless, in absolute terms, larger capacity GF/CF hybrid blade will still have a higher demand for GF and laminate resin in their production, therefore lower impact solutions such as rTP and rGF should still be pursued.

### Circularity of WTB Scenarios

While LCA can evaluate how alternative materials affects specific environmental impact indicators, it is crucial to enhance circularity by diminishing the need for finite virgin raw materials and alleviating the burden on landfills. Ultimately, sustainable solutions for WTB should optimise both circularity and result in an overall reduction in environmental impact across pertinent indicators.

**Fig. 16:**
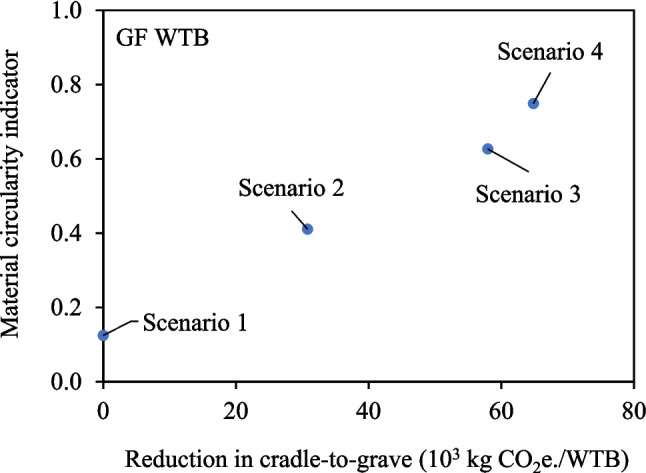
MCI and reduction in GWP (relative to Scenario 1 – Baseline) for each of the scenarios for GF WTB

**Fig. 17:**
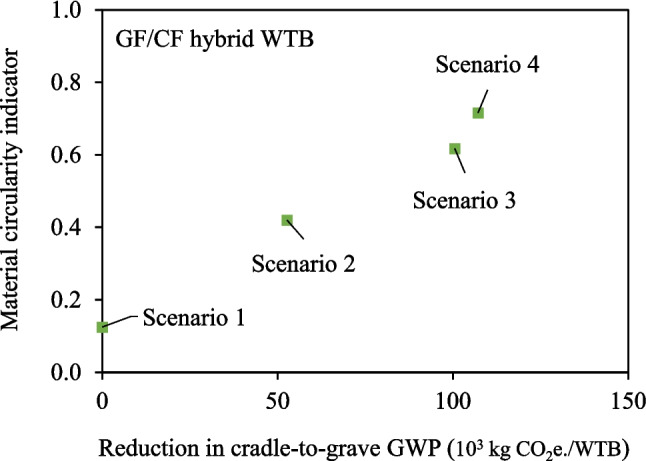
MCI and reduction in GWP (relative to Scenario 1 – Baseline) for each of the scenarios for GF/CF hybrid WTB

**Fig. 18:**
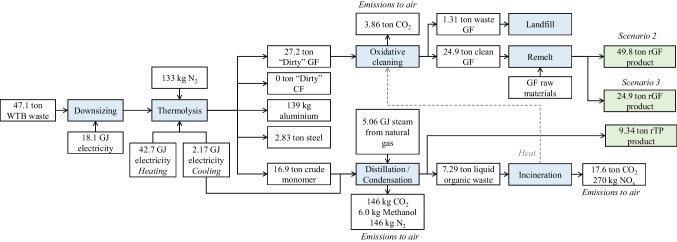
LCI for rGF and rTP production from GF WTB waste feedstock

**Fig. 19:**
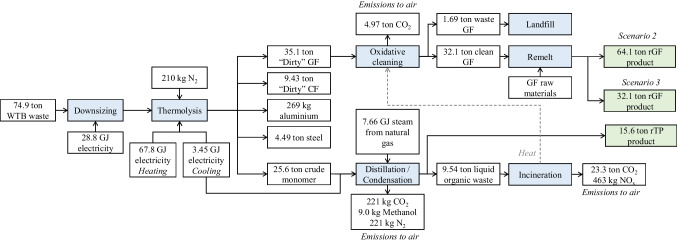
LCI for rGF and rTP production from GF/CF hybrid WTB waste feedstock

Figure 16 and Fig. 17 present the MCI and the reduction in cradle-to-grave GWP for each material and WTB design scenario. The MCI ranges from 0 to 1, with higher values indicating greater circularity, and a fully circular system achieving an MCI of 1. While both Scenarios 1 and 2 involve WTBs made entirely from virgin materials, Scenario 2 WTBs exhibit higher circularity due to the assumption that the TP-based WTB undergoes thermolysis recycling at EoL. However, for WTBs to align with a circular economy, a reliance on virgin or non-restorative materials during production must be addressed.

Figure 16 and Fig. 17 underscore that substituting virgin materials (vGF, vTP, virgin epoxy resin) with restorative rGF and rTP in Scenarios 3 and 4 yields a compounded benefit, by decarbonising WTB production and accelerating the shift towards a closed-loop circular economy for WTB materials. The higher waste GF content in remelted rGF in Scenarios 4 provides the greatest MCI of between 0.72 and 0.75, depending on the WTB design. Achieving a fully circular MCI of 1 necessitates two key actions: 1) incorporating recycled and recyclable and/or bio-based solutions for all other non-restorative materials (core, adhesive, surface coatings, consumables), and 2) mitigating and recycling production waste (both consumable and structural materials).

Certain industry-adopted solutions, like using recycled PET in the production of core kitting and recycling dry fibre offcuts, which have not been considered in the analysis, could theoretically be applied across all scenarios. However, these solutions do not differentiate or prefer one scenario over another. Therefore, the conclusions drawn from Fig. 16 and Fig. 17 remain valid regardless of these considerations. When considering the findings presented, Scenario 4 is the optimal solution (from the scenarios analysed) to simultaneously increase circularity and reduce the environmental impact of typical WTB structures across most environmental indicators.

## Discussion

It is crucial to emphasise that, in contrast to other stages in the WTB lifecycle, the environmental impact of producing a WTB is determined early and remains fixed throughout the lifecycle. This implies that, although ongoing sustainable advancements in blade production are inevitable, the benefits of these advancements cannot be retroactively applied to already deployed WTBs. Conversely, initiatives aimed at mitigating impacts during the WTB operational (e.g., improvements in O&M practices) and EoL phases can have positive effects on WTBs already in operation. While developing effective recycling strategies for EoL WTBs is essential for offsetting impact associated with future virgin material production, the long lifecycle of WTBs means that this approach cannot alleviate the impact of WTBs currently manufactured using environmentally impactful virgin raw materials. In light of the projected exponential growth in global wind energy installations, it is critical that lower impact solutions to the materials used in WTBs are established and their deployment must be a short-term priority.

A potential limitation of using PMMA in WTBs is the dependency on developing recycling processes specifically designed for PMMA. This restricts the feedstock volume available to commercial recyclers employing these technologies, in contrast to alternative recycling methods that are not dependent on specific sources of WTB waste. As of now, there are no operational commercial facilities utilising thermolysis recycling for TP-based WTBs. Therefore, while WTBs in Scenarios 2, 3, and 4 are theoretically recyclable, the practical recycling of these materials is dependent on the future scalability of recycling technologies. Over the next 20–30 years, most decommissioned legacy WTBs will have been produced using epoxy or polyester thermosetting polymers. As a result, the thermolysis recycling technology evaluated may be unsuitable for the majority of WTB waste generated in the near future. Moreover, thermolysis recycling will not be able to produce rTP product from legacy waste made with thermosetting resins.

The utilisation of pyrolysis recycling for thermoset based WTB waste has the capability to produce oil/wax products that may serve as feedstocks for the chemical industry or as substitutes for virgin petrochemicals (Lopez-Urionabarrenechea et al. 2020). However, it is important to note that, unlike PMMA, there is presently no established use case for pyrolysed thermosets derived from WTB waste, and there is a lack of evidence supporting their closed-loop recycling integration back into WTB structures. Recovery of Bisphenol A from standard epoxy systems using targeted catalysed decomposition has been demonstrated (Ahrens et al. 2023), which has the potential to be a drop in solution for epoxy resins used in WTB production (Vestas Wind Systems A/S 2023). This is therefore a potential closed-loop solution to recycling polymers from legacy WTBs which would not be suitable feedstock for recycling in the processes evaluated in this study.

Numerous recycling technologies capable of recovering GF from legacy WTBs or similar composite materials have been demonstrated (Rybicka et al. 2016; Ahrens et al. 2023; Pender and Yang 2020). This implies that a remelt strategy for rGF could potentially be applied to current EoL WTB waste streams if commercially viable technology emerges for extracting clean GF. However, the environmental impact of remelted rGF products is contingent on processes upstream of the remelt phase. Thus, it remains uncertain whether remelted rGF from legacy WTBs reclaimed using alternative recycling technologies will maintain a lower environmental impact than conventional vGF. As an approximate target for technology developers, for remelted rGF to remain a lower GWP solution compared to vGF, the clean GF waste feedstock for remelt must have a GWP of no more than 0.61 kg CO_2_e/kg. The feasibility of achieving this target for other recycling technologies is beyond the scope of this study.

Considering the expected exponential increase in global WTB production and subsequent waste generation in the upcoming decades (Liu and Barlow 2017), the wind energy sector should prioritise the development of low-impact, cost-effective circular solutions for managing existing WTB waste. Additionally, efforts should be directed towards creating the next generation of WTBs that are more readily recycled, thereby enabling the future production of higher-value closed-loop secondary materials. This investigation highlights that the utilisation of TP and remelt strategy for rGF production could contribute to achieving these objectives. Such approaches could not only reduce most environmental impact indicators but also enhance WTB circularity in both the short and long term.

## Conclusions

This study has shown that the production of raw materials used in the WTB structure is the largest contributor to the WTB cradle-to-grave environmental impact. Up to 97% of the GWP attributable to the production of WTBs comes from the upstream production of the raw structural materials. It is therefore critical to identify and deploy lower impact, alternative material solutions in the production of WTBs as means of decarbonisation.

Using LCA, it was determined that rTP and remelted rGF products from EoL WTB waste can reduce most environmental impact indicators compared to virgin counterparts. rTP products are anticipated to exhibit up to 54% lower GWP compared to vTP, with the GWP primarily attributable to GHG emissions during waste incineration. To further reduce rTP GWP, strategies should focus on utilising the non-PMMA fraction of organics to circumvent or mitigate waste incineration. Remelted rGF products are estimated to have 16% to 26% lower GWP than conventional vGF products, which is contingent on the content of waste GF in the melting phase. The main contributor to rGF product GWP is GHG emissions during the remelt phase. Crucially, the GWP of the remelting phase for rGF products remains lower than that of vGF products due to mitigated raw material inputs and less energy-intensive operational conditions during melting.

WTBs constructed with rTP and rGF exhibit lower environmental impacts across most indicators, anticipating a cradle-to-gate GWP reduction of up to 27% compared to WTBs made with virgin epoxy resin and vGF reinforcements. This study underscores the substantial environmental benefits achieved by substituting conventional virgin materials with regenerative rGF and rTP, yields a compounded benefit by decarbonising WTB production and supporting a shift towards a closed-loop circular economy for WTB materials.

## Electronic supplementary material

Below is the link to the electronic supplementary material.

Supplementary file1 (DOCX 73 KB)

## Data Availability

All raw data for results presented in Fig. 6 to Fig. 17 are tabulated in Table S2 to Table S12 in the Supplementary Materials.
