# Supplementary material for: Multi-lifecycle Assessment of Close-loop Recyclable Wind Turbine Blades
Source: Mater Circ Econ. 2026 Jan 26;8(1):5. doi: 10.1007/s42824-025-00211-7 (PMC12835088; doi:10.1007/s42824-025-00211-7)
Supplement: Supplementary file 1 — Supplementary file1 (DOCX 73 KB) [file 42824_2025_211_MOESM1_ESM.docx]

Multi-lifecycle assessment of close-loop recyclable wind turbine blades – Supplementary Materials

# WTB descriptions

Table S1 WTB BoMs, *Scenario 1 & 2 – vGF, Scenario 3 & 4 – rGF; **Scenario 1 – epoxy resin, Scenario 2 – vTP, Scenario 3 & 4 – rTP

| Material type | Proportion of WTB BoM | |
| --- | --- | --- |
|  | GF WTB | GF/CF hybrid WTB |
| Carbon fibre | 0.0% | 12.1% |
| Glass fibre* | 55.6% | 45.1% |
| Laminate resin** | 21.4% | 22.7% |
| PET Core | 10.8% | 7.7% |
| Epoxy adhesive | 5.0% | 5.0% |
| Polyurethane coatings | 0.9% | 1.0% |
| Metallics (steel and aluminium) | 6.3% | 6.4% |

# Description of MCI methodology

MCIs were calculated following the methodology outlined in [52]. The “Product-level Methodology” was used as this was deemed the most suitable approach to assessing material circularity on a single WTB level. In this case, the MCI measures the extent to which linear flow has been minimised and restorative flow maximised for the WTB’s materials, and how long and intensively it is used compared to a similar industry-average product. The MCI is essentially constructed from a combination of three product characteristics: 1) the mass of virgin raw material used in manufacture, 2) the mass of unrecoverable waste that is attributed to the product, and 3) a utility factor that accounts for the length and intensity of the product's use.

The methodology used to calculate the MCI is detailed in [52] and will therefore not be reproduced in this report. A variation on the basic method was used to account for production losses and, when required, additional material inputs to the recycling process (e.g., solvents / chemical inputs in chemical-based recycling processes). The *Multiple Production Steps* approach was also used to account for the different material type inputs used in the blade manufacture [52]. As is common practice within the wind industry, it is assumed that in Scenario 1 and 2 that all raw materials input during the WTB production come from virgin sources. On the other hand, for Scenarios 3 and 4 the rTP and rGF using the WTB production came from restorative sources.

In this work it is assumed the blade life-expectancy and use intensity (e.g., annual energy production) is constant across WTB scenarios analysed. The utility factor was therefore the same across all scenarios and did not influence the MCI calculation. The MCI analysis gives a value between 0 and 1, where higher values indicate higher circularity. A fully circular system has an MCI of 1, which occurs when 1) all feedstock materials are from fully restorative sources, and 2) all waste / output materials are fully restored (e.g., reused, recycled).

Figure 5 Illustrates the input / output materials across WTB lifecycle and the categorisation of restorative / non-restorative flows for each scenario when assessing the MCIs

# Results raw data

Table S2 Raw data Figure 6 – impact per “WTB Baseline GF WTB”

|  | Abiotic Depletion (elements) [kg Sb eq.] | Abiotic Depletion (fossil) [MJ] | Acidification Potential [kg SO2 eq.] | Eutrophication Potential [kg Phosphate eq.] | Freshwater Aquatic Ecotoxicity Pot. [kg DCB eq.] | Global Warming Potential (100 years) [kg CO2 eq.] | Global Warming Potential (100 years), excl biogenic carbon [kg CO2 eq.] | Human Toxicity Potential [kg DCB eq.] | Marine Aquatic Ecotoxicity Pot. [kg DCB eq.] | Ozone Layer Depletion Potential [kg R11 eq.] | Photochem. Ozone Creation Potential [kg Ethene eq.] | Terrestric Ecotoxicity Potential [kg DCB eq.] |
| --- | --- | --- | --- | --- | --- | --- | --- | --- | --- | --- | --- | --- |
| Cradle-to-gate | 0.36967 | 2695826.11260 | 228.54901 | 36.56606 | 1109.54948 | 153678.52290 | 154326.04790 | 11990.54444 | 37724683.74581 | 0.00231 | 23.44150 | 1160.82675 |
| Commissioning | 0.00027 | 307443.46293 | 748.39030 | 83.57045 | 23.67664 | 24383.40167 | 24382.22372 | 1116.89063 | 391242.85427 | 0.00000 | 39.96735 | 4.13901 |
| Use | 0.00194 | 733198.03837 | 718.81517 | 179.27031 | 72.74419 | 53695.54502 | 53690.19883 | 2389.11730 | 903730.22501 | 0.00000 | 50.58407 | 12.17444 |
| Decommissioning | 0.00011 | 129006.96775 | 314.03355 | 35.06716 | 9.93500 | 10231.56805 | 10231.07377 | 468.66072 | 164170.19832 | 0.00000 | 16.77078 | 1.73678 |
| Disposal | 0.00037 | 23370.00504 | 5.44157 | 3.69398 | 7.27694 | 1599.71055 | 1628.61408 | 56.85077 | 197186.34273 | 0.00000 | 0.45820 | 36.14236 |

Table S3 Raw data Figure 7 – impact per “Baseline_GF/CF hybrid WTB”

|  | Abiotic Depletion (elements) [kg Sb eq.] | Abiotic Depletion (fossil) [MJ] | Acidification Potential [kg SO2 eq.] | Eutrophication Potential [kg Phosphate eq.] | Freshwater Aquatic Ecotoxicity Pot. [kg DCB eq.] | Global Warming Potential (100 years) [kg CO2 eq.] | Global Warming Potential (100 years), excl biogenic carbon [kg CO2 eq.] | Human Toxicity Potential [kg DCB eq.] | Marine Aquatic Ecotoxicity Pot. [kg DCB eq.] | Ozone Layer Depletion Potential [kg R11 eq.] | Photochem. Ozone Creation Potential [kg Ethene eq.] | Terrestric Ecotoxicity Potential [kg DCB eq.] |
| --- | --- | --- | --- | --- | --- | --- | --- | --- | --- | --- | --- | --- |
| Cradle-to-gate | 0.72404 | 9202812.03085 | 963.89271 | 143.76423 | 2210.44564 | 546783.32181 | 546554.24853 | 29090.88263 | 66382729.63545 | 0.00537 | 91.63579 | 2233.17045 |
| Commissioning | 0.00027 | 307443.46293 | 748.39030 | 83.57045 | 23.67664 | 24383.40167 | 24382.22372 | 1116.89063 | 391242.85427 | 0.00000 | 39.96735 | 4.13901 |
| Use | 0.00194 | 733198.03837 | 718.81517 | 179.27031 | 72.74419 | 53695.54502 | 53690.19883 | 2389.11730 | 903730.22501 | 0.00000 | 50.58407 | 12.17444 |
| Decommissioning | 0.00011 | 129006.96775 | 314.03355 | 35.06716 | 9.93500 | 10231.56805 | 10231.07377 | 468.66072 | 164170.19832 | 0.00000 | 16.77078 | 1.73678 |
| Disposal | 0.00065 | 43566.45943 | 9.35247 | 7.29437 | 13.55802 | 2962.39815 | 3011.49784 | 100.62092 | 339930.36404 | 0.00000 | 0.80098 | 65.57577 |

Table S4 Raw data Figure 8 – impact per “WTB Baseline GF WTB”

|  | Abiotic Depletion (elements) [kg Sb eq.] | Abiotic Depletion (fossil) [MJ] | Acidification Potential [kg SO2 eq.] | Eutrophication Potential [kg Phosphate eq.] | Freshwater Aquatic Ecotoxicity Pot. [kg DCB eq.] | Global Warming Potential (100 years) [kg CO2 eq.] | Global Warming Potential (100 years), excl biogenic carbon [kg CO2 eq.] | Human Toxicity Potential [kg DCB eq.] | Marine Aquatic Ecotoxicity Pot. [kg DCB eq.] | Ozone Layer Depletion Potential [kg R11 eq.] | Photochem. Ozone Creation Potential [kg Ethene eq.] | Terrestric Ecotoxicity Potential [kg DCB eq.] |
| --- | --- | --- | --- | --- | --- | --- | --- | --- | --- | --- | --- | --- |
| CF production | 0.00000 | 0.00000 | 0.00000 | 0.00000 | 0.00000 | 0.00000 | 0.00000 | 0.00000 | 0.00000 | 0.00000 | 0.00000 | 0.00000 |
| GF production | 0.00975 | 349475.81730 | 67.13579 | 8.93042 | 430.21886 | 32853.51403 | 34745.27851 | 5300.77995 | 28948858.99057 | 0.00231 | 5.47315 | 43.66312 |
| Epoxy resin production | 0.26614 | 1442290.18835 | 79.82388 | 16.43530 | 282.43479 | 73636.97012 | 72473.82760 | 2207.38894 | 2888420.06668 | 0.00000 | 11.25413 | 561.12115 |
| PET core production | 0.00523 | 498862.14713 | 28.33544 | 3.44955 | 103.83104 | 20623.28978 | 20608.96983 | 763.44408 | 1148166.30628 | 0.00000 | 3.77683 | 40.51711 |
| Adhesive production | 0.03130 | 168881.50188 | 9.36916 | 1.92736 | 32.85355 | 8631.76404 | 8497.53470 | 257.58028 | 338617.01355 | 0.00000 | 1.31403 | 64.82134 |
| Coatings production | 0.00499 | 37936.35360 | 5.05832 | 0.62797 | 217.36374 | 1887.26284 | 1954.49688 | 201.00400 | 292040.77508 | 0.00000 | 0.52737 | 209.54139 |
| Metallics production | 0.00057 | 72157.21912 | 19.05042 | 1.70315 | 15.56314 | 7663.57204 | 7665.52008 | 2771.96712 | 3100232.83932 | 0.00000 | 2.20552 | 7.95472 |
| All other cradle-to-gate impacts | 0.05167 | 126222.88522 | 19.77599 | 3.49232 | 27.28436 | 8382.15006 | 8380.42029 | 488.38007 | 1008347.75433 | 0.00000 | -1.10953 | 233.20792 |

Table S5 Raw data Figure 9 – impact per “Baseline_GF/CF hybrid WTB”

|  | Abiotic Depletion (elements) [kg Sb eq.] | Abiotic Depletion (fossil) [MJ] | Acidification Potential [kg SO2 eq.] | Eutrophication Potential [kg Phosphate eq.] | Freshwater Aquatic Ecotoxicity Pot. [kg DCB eq.] | Global Warming Potential (100 years) [kg CO2 eq.] | Global Warming Potential (100 years), excl biogenic carbon [kg CO2 eq.] | Human Toxicity Potential [kg DCB eq.] | Marine Aquatic Ecotoxicity Pot. [kg DCB eq.] | Ozone Layer Depletion Potential [kg R11 eq.] | Photochem. Ozone Creation Potential [kg Ethene eq.] | Terrestric Ecotoxicity Potential [kg DCB eq.] |
| --- | --- | --- | --- | --- | --- | --- | --- | --- | --- | --- | --- | --- |
| CF production | 0.06765 | 5119948.41263 | 636.72780 | 89.56073 | 662.19305 | 319128.20073 | 319118.51496 | 12389.01640 | 22745356.48356 | 0.00000 | 57.33911 | 242.07662 |
| GF production | 0.01254 | 336637.44296 | 64.67263 | 8.60549 | 414.41744 | 31646.60731 | 33468.87574 | 5106.05313 | 27885390.90551 | 0.00537 | 5.27523 | 42.06225 |
| Epoxy resin production | 0.44889 | 2428656.74979 | 134.53443 | 27.69081 | 474.42387 | 124046.95571 | 122098.88473 | 3712.23787 | 4865945.53574 | 0.00000 | 18.93063 | 940.14207 |
| PET core production | 0.00596 | 568336.13132 | 32.28157 | 3.92996 | 118.29106 | 23495.39005 | 23479.07584 | 869.76504 | 1308065.56556 | 0.00000 | 4.30281 | 46.15972 |
| Adhesive production | 0.04973 | 268296.66815 | 14.88449 | 3.06194 | 52.19339 | 13713.00886 | 13499.76298 | 409.20959 | 537950.07448 | 0.00000 | 2.08755 | 102.97961 |
| Coatings production | 0.00949 | 71381.68165 | 9.51892 | 1.24742 | 403.83125 | 3546.48776 | 3677.81573 | 375.25333 | 545316.79780 | 0.00000 | 0.98973 | 389.19313 |
| Metallics production | 0.00107 | 119708.17476 | 32.15030 | 2.82193 | 27.24296 | 12619.61378 | 12623.42744 | 5171.26565 | 5893223.69167 | 0.00000 | 3.62441 | 13.74137 |
| All other cradle-to-gate impacts | 0.12871 | 289846.76959 | 39.12256 | 6.84595 | 57.85263 | 18587.05762 | 18587.89111 | 1058.08160 | 2601480.58115 | 0.00000 | -0.91369 | 456.81569 |

Table S6 Raw data Figure 10 – impact per kg of TP

|  | Abiotic Depletion (elements) [kg Sb eq.] | Abiotic Depletion (fossil) [MJ] | Acidification Potential [kg SO2 eq.] | Eutrophication Potential [kg Phosphate eq.] | Freshwater Aquatic Ecotoxicity Pot. [kg DCB eq.] | Global Warming Potential (100 years) [kg CO2 eq.] | Global Warming Potential (100 years), excl biogenic carbon [kg CO2 eq.] | Human Toxicity Potential [kg DCB eq.] | Marine Aquatic Ecotoxicity Pot. [kg DCB eq.] | Ozone Layer Depletion Potential [kg R11 eq.] | Photochem. Ozone Creation Potential [kg Ethene eq.] | Terrestric Ecotoxicity Potential [kg DCB eq.] |
| --- | --- | --- | --- | --- | --- | --- | --- | --- | --- | --- | --- | --- |
| vTP | 0.00000 | 97.58444 | 0.00820 | 0.00089 | 0.02549 | 3.79583 | 3.79174 | 0.12632 | 132.49349 | 0.00000 | 0.00076 | 0.01121 |
| rTP_GF WTB | 0.00000 | 4.09659 | 0.01500 | 0.00383 | 0.00043 | 2.14942 | 2.14943 | 0.04465 | 31.46870 | 0.00000 | 0.00093 | 0.00040 |
| rTP_GF/CF hybrid WTB | 0.00000 | 3.70356 | 0.01529 | 0.00391 | 0.00039 | 1.72966 | 1.72967 | 0.04453 | 28.44563 | 0.00000 | 0.00094 | 0.00036 |

Table S7 Raw data Figure 11 – GWP (kg CO2e.) per kg of TP

|  | vPMMA | Downsizing | Thermolysis | Distillation | Waste incineration | Total |
| --- | --- | --- | --- | --- | --- | --- |
| vTP | 3.796 | / | / | / | / | 3.796 |
| rTP_GF WTB | / | 0.018 | 0.148 | 0.055 | 1.887 | 2.107 |
| rTP_GF/CF hybrid WTB | / | 0.016 | 0.134 | 0.050 | 1.492 | 1.692 |

Table S8 Raw data Figure 12 – impact per kg of GF

|  | Abiotic Depletion (elements) [kg Sb eq.] | Abiotic Depletion (fossil) [MJ] | Acidification Potential [kg SO2 eq.] | Eutrophication Potential [kg Phosphate eq.] | Freshwater Aquatic Ecotoxicity Pot. [kg DCB eq.] | Global Warming Potential (100 years) [kg CO2 eq.] | Global Warming Potential (100 years), excl biogenic carbon [kg CO2 eq.] | Human Toxicity Potential [kg DCB eq.] | Marine Aquatic Ecotoxicity Pot. [kg DCB eq.] | Ozone Layer Depletion Potential [kg R11 eq.] | Photochem. Ozone Creation Potential [kg Ethene eq.] | Terrestric Ecotoxicity Potential [kg DCB eq.] |
| --- | --- | --- | --- | --- | --- | --- | --- | --- | --- | --- | --- | --- |
| vGF | 0.00000 | 13.56239 | 0.00261 | 0.00035 | 0.01670 | 1.27497 | 1.34839 | 0.20571 | 1123.44140 | 0.00000 | 0.00021 | 0.00169 |
| rGF_50%_GF WTB | 0.00000 | 12.03199 | 0.00228 | 0.00029 | 0.00952 | 1.07317 | 1.09675 | 0.15330 | 1084.64179 | 0.00000 | 0.00018 | 0.00126 |
| rGF_100%_GF WTB | 0.00000 | 10.51654 | 0.00195 | 0.00024 | 0.00236 | 0.94722 | 0.92090 | 0.10107 | 1046.07018 | 0.00000 | 0.00015 | 0.00084 |
| rGF_50%_GF/CF hybrid WTB | 0.00000 | 12.03155 | 0.00228 | 0.00029 | 0.00952 | 1.07314 | 1.09672 | 0.15330 | 1084.63707 | 0.00000 | 0.00018 | 0.00126 |
| rGF_100%_GF/CF hybrid WTB | 0.00000 | 10.51566 | 0.00195 | 0.00024 | 0.00236 | 0.94716 | 0.92083 | 0.10106 | 1046.06073 | 0.00000 | 0.00015 | 0.00084 |

Table S9 Raw data Figure 13 – GWP (kg CO2e.) per kg of TP

|  | vGF | Downsizing | Thermolysis | GF cleaning | Remelt | Waste disposal | Total |
| --- | --- | --- | --- | --- | --- | --- | --- |
| vGF | 1.27497 | 0.00000 | 0.00000 | 0.00000 | 0.00000 | 0.00000 | 1.27497 |
| rGF_50% | 0.00000 | 0.01802 | 0.04473 | 0.07751 | 0.93231 | 0.00060 | 1.07317 |
| rGF_100% | 0.00000 | 0.03605 | 0.08946 | 0.15501 | 0.66551 | 0.00119 | 0.94722 |

Table S10 Raw data Figure 14 – impact per “GF WTB”

|  | Abiotic Depletion (elements) [kg Sb eq.] | Abiotic Depletion (fossil) [MJ] | Acidification Potential [kg SO2 eq.] | Eutrophication Potential [kg Phosphate eq.] | Freshwater Aquatic Ecotoxicity Pot. [kg DCB eq.] | Global Warming Potential (100 years) [kg CO2 eq.] | Global Warming Potential (100 years), excl biogenic carbon [kg CO2 eq.] | Human Toxicity Potential [kg DCB eq.] | Marine Aquatic Ecotoxicity Pot. [kg DCB eq.] | Ozone Layer Depletion Potential [kg R11 eq.] | Photochem. Ozone Creation Potential [kg Ethene eq.] | Terrestric Ecotoxicity Potential [kg DCB eq.] |
| --- | --- | --- | --- | --- | --- | --- | --- | --- | --- | --- | --- | --- |
| Scenario 1 - Baseline | 0.37236 | 3888844.58670 | 2015.22960 | 338.16796 | 1223.18226 | 243588.74818 | 244258.15830 | 16022.06387 | 39381013.36614 | 0.00231 | 131.22191 | 1215.01935 |
| Scenario 2 - vTP/vGF | 0.12683 | 3557013.01527 | 2022.80233 | 328.45515 | 1228.75103 | 212799.11385 | 214497.25253 | 15230.95836 | 37798509.61021 | 0.00231 | 128.38532 | 769.95443 |
| Scenario 3 - rTP/rGF_50% | 0.11106 | 2453578.32991 | 2085.79667 | 359.40943 | 761.27205 | 185632.97472 | 186092.24207 | 12895.83901 | 35430218.91393 | 0.00148 | 129.18469 | 635.14597 |
| Scenario 4 - rTP/rGF_100% | 0.10670 | 2391724.08149 | 2073.18031 | 357.70278 | 573.36438 | 178757.96286 | 177930.86549 | 11474.62875 | 34192939.15122 | 0.00065 | 128.11146 | 620.78658 |

Table S11 Raw data Figure 15 – impact per “GF/CF hybrid WTB”

|  | Abiotic Depletion (elements) [kg Sb eq.] | Abiotic Depletion (fossil) [MJ] | Acidification Potential [kg SO2 eq.] | Eutrophication Potential [kg Phosphate eq.] | Freshwater Aquatic Ecotoxicity Pot. [kg DCB eq.] | Global Warming Potential (100 years) [kg CO2 eq.] | Global Warming Potential (100 years), excl biogenic carbon [kg CO2 eq.] | Human Toxicity Potential [kg DCB eq.] | Marine Aquatic Ecotoxicity Pot. [kg DCB eq.] | Ozone Layer Depletion Potential [kg R11 eq.] | Photochem. Ozone Creation Potential [kg Ethene eq.] | Terrestric Ecotoxicity Potential [kg DCB eq.] |
| --- | --- | --- | --- | --- | --- | --- | --- | --- | --- | --- | --- | --- |
| Scenario 1 - Baseline | 0.72701 | 10416026.95932 | 2754.48420 | 448.96652 | 2330.35950 | 638056.23470 | 637869.24269 | 33166.17220 | 68181803.27710 | 0.00537 | 199.75897 | 2316.79646 |
| Scenario 2 - vTP/vGF | 0.30757 | 9847060.29738 | 2767.65409 | 431.41284 | 2338.97860 | 585338.65553 | 586909.45221 | 31816.36505 | 65492103.46097 | 0.00537 | 194.91158 | 1552.82596 |
| Scenario 3 - rTP/rGF_50% | 0.28232 | 8000827.94300 | 2890.37792 | 487.17808 | 1679.86551 | 537520.09246 | 537931.51694 | 28887.00582 | 62314889.81202 | 0.00316 | 197.22785 | 1332.54649 |
| Scenario 4 - rTP/rGF_100% | 0.27672 | 7941245.97683 | 2878.22363 | 485.53272 | 1498.85943 | 530897.64030 | 530069.95608 | 27518.00386 | 61123062.87712 | 0.00096 | 196.19264 | 1318.71320 |

Table S12 Raw data Figure 16 and Figure 17

|  | GF WTB (Figure 16) | | | | GF/CF hybrid WTB (Figure 17) | | | |
| --- | --- | --- | --- | --- | --- | --- | --- | --- |
|  | Scenario 1 | Scenario 2 | Scenario 3 | Scenario 4 | Scenario 1 | Scenario 2 | Scenario 3 | Scenario 4 |
| GWP per blade (kg CO2e.) | 243588.74818 | 244258.15830 | 16022.06387 | 39381013.36614 | 638056.23470 | 637869.24269 | 33166.17220 | 68181803.27710 |
| MCI | 0.12461 | 0.41073 | 0.62675 | 0.74884 | 0.12474 | 0.41992 | 0.61712 | 0.71575 |
